# Cytological physiognomies and genotype distribution of human papillomaviruses among HPV/HIV co-infected and HPV mono-infected women

**DOI:** 10.4314/ahs.v21i1.33

**Published:** 2021-03

**Authors:** Lucy Wanja Karani, Stanslaus Musyoki, Robert Orina, Christopher Khayeka-Wandabwa, Benuel Nyagaka

**Affiliations:** 1 School of Health Science, Kisii University, Kenya; 2 School of Pharmaceutical Science and Technology (SPST), Health Science Platform, Tianjin University, Tianjin 300072, China

**Keywords:** Human immunodeficiency virus (HIV), Human Papillomavirus (HPV), co-infection, genotype, cytology

## Abstract

**Background:**

Co-infection of High Risk Human Papillomavirus (HR-HPV) and HIV is thought to favour initiation of intraepithelial squamous cell lesion and subsequent progression to cervical carcinoma.

**Objectives:**

Evaluation of cytological physiognomies in relation to possible age influence and the genotype distribution of human papillomaviruses among HPV/HIV co-infected and HPV monoinfected women in Kisii, Kenya.

**Methods:**

The case-control study enrolled 42 HPV/HIV co-infected and 42 HPV monoinfected women. Cervical swabs were collected in ThinPrep vials for HPV tying and cytological analysis. HPV subtypes were assayed by Xpert® HPV system (GXHPV-CE-10).

**Results:**

Mono-infected women aged 30–39 years had the highest proportion of low grade squamous intraepithelial lesion (LSIL) at 14 (16.67%) while the co-infected aged 50–59 years had the highest proportion of high grade squamous intraepithelial lesion (HSIL) at 9 (10.71%). HPV-16 genotype was the most predominant and it increased with age rise. Older coinfected and mono-infected women (>40 years) had HSIL and LSIL as the most predominant cytological grade respectively.

**Conclusion:**

The predominance of HPV-16 and HPV-18/45 genotypes in the study setting is a consideration that would benefit targeted prophylactic vaccination programs. HPV testing and cervical cancer screening for young and older women on a regular basis ought to be reinforced.

## Introduction

Immunosuppression due to Human Immunodeficiency Virus (HIV) is associated with greater prevalence and broader range of the high risk human papilloma virus (HR-HPV) genotypes in women with cervical cancer 1, 2. Data from Kenya Medical Research Institute (KEMRI) which serves as regional cancer registry approximates 80% of reported cases of cervical carcinoma are diagnosed at advanced stages, when little can be achieved in terms of curative treatment 3, 4. Globally, cervical cancer remains largely preventable by screening and prophylactic vaccination 3, 4. The vaccination targets specific HPV types despite differences in HPV type prevalence in different geographical regions among other factors that tend to influence HPV types in a population^5^, ^6^. There are currently three types of vaccines already approved for use by FDA namely Cervarix which is designed as a 2-valent vaccine targeting the antigens of HPV 16/18, Gardasil designed as a 4-valent vaccine that targets high risk HPV 16/18 and low risk HPV 6 and 11 while Gardasil 9 is a 9-valent vaccine that targets the antigen of HPV 6,11,16,18 31,33,45,52 and 58 4.

Infection with high risk HPV types is closely associated with intraepithelial neoplasia progressively resulting in cervical carcinoma 7. Two common HPV types namely 16 and 18 have been associated with upto 70% of all known cases of cervical carcinoma worldwide 8–10 while high risk HPV types distribution in different geographical populations in Kenya seem to vary 9. To predict the impact of current vaccines and for the improvement of screening programs, regional and localized data on distribution of HPV types in women at risk is crucial 11, 12. In Kenya, HPV data in women infected with HIV is scarce despite the fact that such data in comorbidity is likely to be different from that found in monoinfections cytology trends 2, 9, 13. Presence of HIV is thought to increase the risk and susceptibility to infections with oncogenic associated human papilloma virus (HPV) which subsequently accelerates the natural history of Invasive Cervical Carcinoma (ICC)^13^. Some studies indicate that bruises at the genital regions and other forms of inflammations increases the risk of acquiring HIV and therefore promoting chances of cancer progression when HPV infects 14. Yet, the presence of the oncogenic HPV types in co-joint infections of HPV/HIV is thought to favour greatly induction of intraepithelial squamous lesion and initiation of cervical carcinoma with woman age being crucial. Cytological studies indicate that there is a greater rate of high-risk HPV type persistence in HIV infected women and these are strongly associated with a greater risk of progression to high-grade squamous intraepithelial lesions (HSIL) that eventually result to invasive cervical carcinoma^15^. The present study aimed to evaluate cytological physiognomies in relation to possible age influence in genotype distribution of human papillomaviruses among HPV/HIV+ co-infected and HPV monoinfected women respectively in Kisii, Kenya.

## Materials and methods

### Study setting, sample size and sample collection

In the case-control study conducted at Kisii Teaching and Referral Hospital (KTRH) located in Kisii County Kenya, participants enrollment inclusion and exclusion criteria was essentially carried out as previously described 16. Thus, n=42 as HPV/HIV co-infected cases and n=42 as HPV monoinfected controls were consented and enlisted 16. The study adopted the double proportion formula for sample size determination^16^,^17^. The research protocol was approved by the Scientific Ethical Committee of University of Eastern Africa Baraton and National Commission for Science, Technology and Innovation (NACOSTI). Further research authorization was given from the office of County Director of Education Kisii County. Voluntary informed consent was sought from each study participant prior to the interviews and sample collection.

Briefly, in the one stop sample collection, consented women were recruited into the study. HIV testing was then done according Ministry of Health, National AIDS & STI Control Program (NASCOP) 2018 national testing guidelines using Determine® rapid test kit (Abbot Pharmaceuticals, Chicago, USA), and the positive results confirmed by Uni-Gold® (Trinity Biotech Plc, Ireland). There after the participants underwent a preliminary visual inspection with acetic acid (VIA) prior to cervical samples collection with a cyto-broom. Te Pap smears slides were prepared in duplicate for Papanicolaou (Pap) staining. Three independent pathologists read the slides. The cells were classified according to the revised standardized Bethesda classification^18^–^20^. Women with Pap smear abnormalities were referred to the department of Obstetrics & Gynecology where clinical follow up and routine management was done. The cervical samples were also preserved in thin-prep vials for HPV typing. In HR-HPV genotyping, all samples were analysed by Xpert® HPV assay system (GXHPV-CE-10) as described 16. In every assay, a Probe Check Control (PCC) and a Sample Adequacy Control (SAC) were included to ensure quality control. All HR-HPV types were pooled in different paths indicated as P1, P2, P3, P4 and P5 as follows; P1 colour channel HPV 16, P2 colour channel for HPV 18/45 pooled result, P3 for the pooled result of any of HPV types 31, 33, 35,52, 58, P4 for the pooled result of either of HPV types 51,59 and P5 colour channel for the pooled result of any of HPV types 39, 56, 66 or 68 16.

### Data analysis

Descriptive statistics was applied for cervical lesions and HPV characterization and the significance of their difference tested by chi-square statistics. Significance level was set at P < 0.05.

## Results

**Figure 1:**
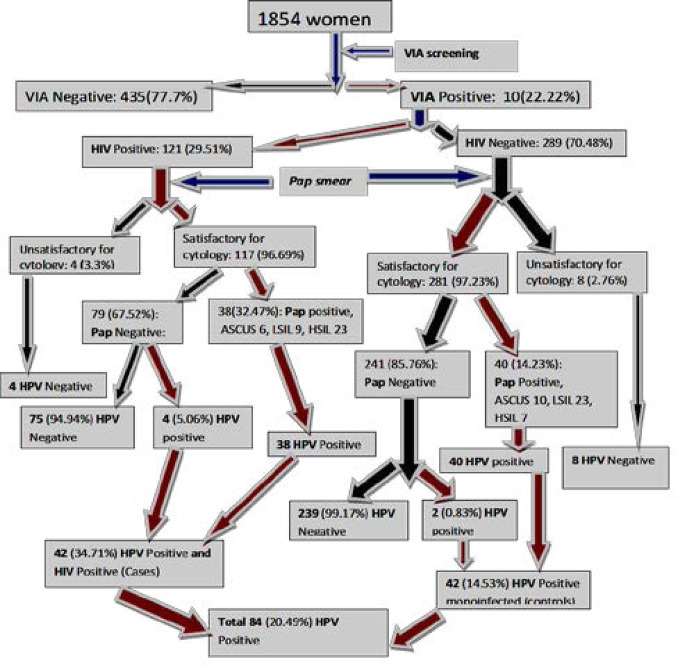
Recruitment process from preliminary to desired sample size

The enrolment of participants was consecutive and purposive which recruited 1854 consented participants. The women first underwent Visual Inspection with 5% Acetic Acid (VIA). There were 410 (22.22%) VIA positive women while 1435 (77.7%) were VIA negative. All the 410 VIA positive samples qualified for cytological analysis and HPV testing. The 121 VIA/HIV positive women had cervical cell exfoliated and smears prepared for cytological evaluation. A portion of the cervical sample was preserved for HPV characterization. Out of the 121 samples, 4 (3.3%) samples/slides were classified as unsatisfactory for cytology while 117 (96.69%) were satisfactory. The satisfactory samples had 79 (67.52%) women with normal cytology and 38 (32.47%) with abnormal cytology. There were 289 VIA positive/HIV negative women. These 289 women had cervical cell samples exfoliated and smears prepared for cytological evaluation. Out of 289 Pap smear samples, 8 (2.76%) were classified as unsatisfactory for cytology while 281 (97.23%) were satisfactory for cytology. The satisfactory samples had 241 (85.76%) women with normal cytology while 40 (14.23%) had abnormal cytology. All the unsatisfactory samples from VIA/HIV positive 4 (3.3%) and those of VIA positive/HIV negative 8 (2.76) were also HPV negative. Abnormal cytology had 38 (32.47%) VIA/HIV positive and 40 (14.23%) VIA positive/HIV negative women who were HPV positive. Only 4 (5.06%) with normal cytology from the VIA/HIV positive and 2 (0.83%) of VIA positive/HIV negative were HPV positive. In total 42 (34.71%) of HIV positive were HPV positive (cases) while 42(14.53%) of HIV negative were HPV positive (controls). The summary of the recruitment process is in Figure 1.

Among the women recruited, age categories between HPV/HIV co-infected women (cases) and those in HPV monoinfected women (controls) group were analysed. The highest proportion of cases were in the upper age limit (50 -59 years), with 14 (33.33%) cases. Controls in this age limit were only 5 (11.90%). The highest proportion of controls was in the age category 30 – 39 years where there were 10 (23.81%) cases compared to 15 (35.71%) controls in this age category. Frequencies of the cases and controls in different age categories are represented in Table 1.

**Table 1:** Frequencies of women in different age categories by HIV status

Category	HPV/HIV+VE (Cases), n (%)	HPV/HIV–VE (Controls), n (%)
< 29 Years	10 (23.81)	12 (28.57)
30 – 39 Years	10 (23.81)	15 (35.71)
40 – 49 Years	8 (19.05)	10 (23.81)
50 – 59 Years	14 (33.33)	5 (11.90)

The distribution of women required to recruit the adequate sample size of cases (121) compared to that of controls (289), with controls requiring more than twice the population of cases is summarized in Table 2.

**Table 2:** Recruitment summary of cases and controls from the 410 VIA positive women

HIV Status	HPV/HIV+VE	HPV/HIV– VE	Total
HIV positive (HIV+VE)	42	79	121
HIV negative (HIV-VE)	42	247	289

Total women	84	326	410

HPV/HIV+VE denotes HPV/HIV positive (Cases) & HPV/HIV–VE denotes HPV/HIV negative (Controls)

Overall mean age for the participants was 37.79 ± 10.21 years. 42 HPV/HIV co-infected women (cases) had mean age of 40.36 ± 11.318 years while 42 HPV mono-infected women (controls) had mean age of 35.21 ± 9.495 years.

Prevalence of cytological grades in the study population The overall cytological categories in both co-infection (HPV/HIV) and mono-infections (HPV) combined were classified according to the revised standardized Bethesda classification^18^, ^19^. These included atypical squamous cell of undetermined significance (ASCUS), low grade intraepithelial lesions (LSIL) and high grade intraepithelial lesions (HSIL). Normal cytology had 6 (7.1%), ASCUS had 16 (19.0%), LSIL had 32 (38.1%) and HSIL had 30 (37.5%) women. The most common lesions were LSIL, followed by HSIL, ASCUS while normal cytology had the least proportion.

### Cytological characterization by age

**Figure 2:**
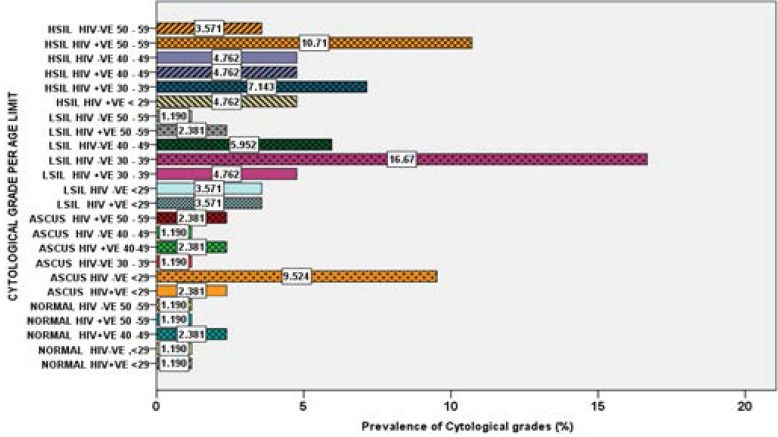
Prevalence of cytological categories in HPV+/HIV+ cases and HPV+ /HIV- women control groups by age

The categorization of cytological grades was done according to the ages of women and the trends presented in Figure 2. Normal cytology was found in equal proportion in both cases and controls 1 (1.2%) in the <29 age category. All women in the control group in the age category 40–49 years, had abnormal cytology while cases had 2 (2.4%) of the women with normal cytology. In the age category 50–59 years, both cases and controls had 1(1.2 %) women with normal cytology. Cases falling in the ages categories <29, 30–39, 40–49 and 50–59 years had 2(2.4%), 0(0.0%) 2(2.4%), 2(2.4%) ASCUS cytological category respectively, compared to controls who had, 8(9.5%), 1(1.2%), 1(1.2%), 0(0.0%) prevalence of ASCUS cytological category respectively. In cases the LSIL cytological category had 3(3.6%), 4(4.8%), 0(0.0%), 2(2.4%) prevalence in the ages categories <29, 30–39, 40–49 and 50–59 years respectively, compared to controls who had, 3(3.6%), 14(16.7%), 5(6.0%), 1(1.2%) prevalence respectively. In cases the HSIL cytological category had 4(4.8%), 6(7.1%), 4(4.8%), 9(10.7%) prevalence in the age (years) categories <29, 30–39, 40–49 and 50–59 in the order, compared to controls who had, 0(0.0%), 0(0.0%), 4(4.8%), 3(3.6%) prevalence correspondingly. The greatest prevalence 14 (16.67%) was that of LSIL observed in HIV negative between 30–39 years followed by that of HSIL in HIV positive women 9 (10.71%) between 50–59 years. The prevalence of cytological grades in the cases were significantly different from those of controls (p = 0.109).

### HPV genotypes characterization by age

**Figure 3:**
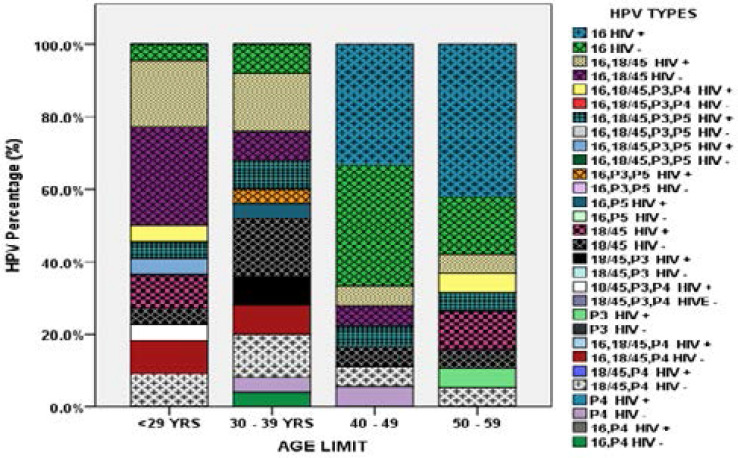
HPV type by age categories among women in cases and control groups. P1 colour channel HPV 16, P2 colour channel for HPV 18/45 pooled result, P3 for the pooled result of any of HPV types 31, 33, 35, 52, 58, P4 for the pooled result of either of HPV types 51, 59 and P5 colour channel for the pooled result of any of HPV types 39, 56, 66 or 68

The prevalence of HPV types was done in the four age brackets namely; >29, 30–39, 40–49 and 50–59 years (Figure 3). In the age bracket >29 years, cases had a total prevalence of 10 (11.9%) compared to controls who had 12 (14.3%). In this age bracket the HPV type category (16, 18/45) had the highest prevalence of 4 (4.8%) and 6 (7.1%) in cases and controls respectively. In this age bracket, type category (16, 18/45) of the controls had the highest prevalence of 6 (7.1%). The cases followed with a prevalence of 4 (4.8%) in the HPV type category (16, 18/45). The second most prevalent type category was (18/45) in cases, (16, 18/45, P4) and (18/45, P4) in controls with a prevalence of 2 (2.4%) each. All the other type combinations (18/45, P3, P4), (16, 18/45, P3, P5), (16, 18/45, P3, P4), (16, 18/45, P3) in cases and type 16 in controls had the same prevalence of 1(1.2%). In the age bracket 30 – 39 years, the total prevalence of HPV infections in cases and controls was 10 (11.9%) and 15 (17.9%) respectively whereas HPV types categories (16, 18/45) in cases and (18/45) in controls had the highest prevalence of 4 (4.8%) each. The HPV types category (18/45, P4) followed with a prevalence of 3 (3.6%) in controls. Type categories (18/45, P3) and (16, 18/45, P3, P4) in cases, (16), (16, 18/45), (16, P4) in controls had the same prevalence of 2 (2.4%). All the other type combinations (16) and (16, P3, P5) in cases as well as (16, P4) and (P4) in controls had a prevalence of 1(1.2%) in this age category. In age bracket 40–49 year, the total prevalence of HPV infections was 8 (9.5%) and 10 (11.9%) in cases and controls respectively. The highest HPV prevalence 6 (7.1%) in this age bracket was in types 16 for both cases and controls. All the other type combinations (16, 18/45) and (16, 18/45, P3, P4) in cases as well as (1618/45), (18/45), (18/45, P4), (P4) in controls had a prevalence of 1(1.2%). In the age bracket 50–59 year the total prevalence of HPV infections was 14 (16.6%) and 5 (6.0%) for cases and controls respectively. The highest HPV prevalence in this age category was 8(9.5% exhibited in types 16 of cases followed by same type with a prevalence of 3 (3.6%) in controls. Type (18/45) had a prevalence of 2(2.4%) in cases. All the other type combinations (16, 18/45), (16, 18/45, P3), (16, 18/45, P3, P4) and (P3) in cases as well as (18/45) and (18/45, P4) in controls had the same prevalence of 1(1.2%) in this age category.

## Discussion

The most prevalent HR-HPV subtype was HPV 16 which was consistent with other settings 21, 22 observations whereas its prevalence increased with increase in age. The ratio of HIV positive to HIV negative screened to attain the required sample size was approximately 1:2 for women aged 18–59 years. This study indicates a greater burden of HPV in HIV positive compared to HIV negative similar to findings from sub-Saharan city of Cape Town, where ratio of HPV in HIV positive to HIV negative women was1:5 in women between 17 and 65 years 23. The present study participants had an overall a mean age of 37.79 years with a mean age of 40.36 and 35.21 years for the HPV/HIV co-infected and HPV mono-infected women respectively. The age trend was comparable to the Thika, central Kenya HPV project. The Thika project reported type specific prevalence of HPV in 498 women with mean age of 36 years while the recruited women were 18 to 74 years of age 24 noting the fact that differences in prevalence of HPV in women who are already in menopausal age is influenced by immunosenescence which increases with increase in age 25. In the north rift region of Kenya, further findings showed that age of the participant, was among the factors that increased the severity of dysplasia and HPV prevalence in women 26 findings that resonates with the fact that participants' age affect the prevalence of HPV^25^ which is in agreement with the current study.

HPV characterization in the current study population showed that HPV type 16 had the highest prevalence of 26 (20.8%) followed by HPV types combination (16, 18/45) with 19 (22.6%). HR-HPV type 16 had a higher prevalence of 4 (4.8%) in all ages compared to HPV type (18/45) which had 2 (2.4%) in this population. A similar dimension was observed in the study conducted in the Central Kenya at Thika where the prevalence of HPV type 16 was 4.6% 24 with further high prevalence at all ages noted in HPV type 52, 56, 66, and 18 24. In the current study, HPV 16 had the highest prevalence 12 (14.29 %) in the ages between 40–49 years followed by 11 (13.1%) in the ages between 50–59 years. Gheda et al evaluated 24 HR-HPV types and the established prevalence of HR-HPV was 33.1% with type 16 and 42 being the most predominant at 6.7% and 6.8% respectively 27. Reporting based on United Kingdom (UK) research pointed to HPV types 16 and 18 to be high (83.0%) in cervical cancer cases and most prevalent in the younger women but then decreased with age 28. Studies in Uganda showed a greater prevalence in high risk HPV types in women above 40 years which is comparable to what was observed in the current study population 29. Presented findings had no appreciable variance from what was observed in a study in Tanzania where lesions increased with persistence of high risk HPV types 15. In cervical intraepithelial neoplasia stage 3(CIN3) the two HPV types 16 and 18 had a prevalence of (77.2%) in the younger women aged <30 years which decreased with age. Equally, other HPV types 31, 33, 45, 52 and 58 in this study had a prevalence of 16.1% and showed no association to age 28. McDonald et al findings reports on the predominant HR-HPV types as 16, 35, and 58 while type distribution was similar despite HIV status with HPV type 18 more commonly found in older women between 40 to 65 years infected with HIV but the younger women between 17 to 29 years were less commonly affected by that type 23. The observed findings trends are in concurrence with the present research in that HR-HPV type 16 is a predominant genotype in the elderly women but also affect the other ages. Bangkok Metropolitan women findings in Thailand established that HPV type 16, 52, 58 18 and 51 were the most common types with a prevalence of (1.4%), (1.0%), (0.9%), (0.7%) and (0.7%) respectively 30 whereas finding from Kenya from north rift town of Eldoret closely relating to the current study establishing the most prevalent HPV as HPV type 52 26. Despite both Eldoret and Kisii being in Kenya, they differ in the most prevalent HPV type.

The presented findings had some limitations. The current study was not within reach to evaluate socio-demographic determinants where differences in sexual behaviour and population genetics in a geographical area or ethnicity 31 would play a role as confounding factors in HPV/HIV comorbidities. Also, while the relatively small sample size limits the generalization of the study outcomes, the findings will serve as a good basis for a detailed investigation of evaluation of cytological and genotype trends in HPV infections and comorbidities as well as the associated socio-demographic determinants in women population in south western Kenya and beyond.

## Conclusion

Low grade lesions were predominant in women aged < 29 with high proportions of HSIL in coinfected and LSIL in monoinfected women aged >40 years and <40 years respectively. Targeted prophylactic vaccines should incorporate HPV-16 and HPV-18/45, due to predominant infection rates in the women in south western Kenya while HPV testing and cervical cancer screening for young and older on a regular basis ought to be reinforced. There was a higher prevalence of HRHPV types in high grade lesions in co-infections which were double that of mono-infections.
